# The Spectrum of Neurological and White Matter Changes and Premutation Status Categories of Older Male Carriers of the *FMR1* Alleles Are Linked to Genetic (CGG and FMR1 mRNA) and Cellular Stress (AMPK) Markers

**DOI:** 10.3389/fgene.2018.00531

**Published:** 2018-11-12

**Authors:** Danuta Z. Loesch, Nicholas Trost, Minh Q. Bui, Eleanor Hammersley, Sui T. Lay, Sarah J. Annesley, Oana Sanislav, Claire Y. Allan, Flora Tassone, Zhi-Ping Chen, Kevin R. W. Ngoei, Bruce E. Kemp, David Francis, Paul R. Fisher, Elsdon Storey

**Affiliations:** ^1^Department of Psychology and Counselling, School of Psychology and Public Health, College of Science Health and Engineering, La Trobe University, Melbourne, VIC, Australia; ^2^Medical Imaging Department, St Vincent’s Hospital, Melbourne, VIC, Australia; ^3^Centre for Epidemiology and Biostatistics, Melbourne School of Population and Global Health, University of Melbourne, Melbourne, VIC, Australia; ^4^Discipline of Microbiology, Department of Physiology Anatomy and Microbiology, School of Life Sciences, College of Science Health and Engineering, La Trobe University, Melbourne, VIC, Australia; ^5^UC Davis MIND Institute, Sacramento, CA, United States; ^6^St Vincent’s Institute of Medical Research, Melbourne, VIC, Australia; ^7^Cytomolecular Diagnostic Research, Victorian Clinical Genetics Services, Melbourne, VIC, Australia; ^8^Department of Medicine (Neuroscience), Monash University, Melbourne, VIC, Australia

**Keywords:** *FMR1* premutation, CGG repeats, *FMR1* mRNA, AMPK kinase, cellular stress, motor scores, cognitive status, white matter hyperintensities

## Abstract

The fragile X premutation (PM) allele contains a CGG expansion of 55–200 repeats in the *FMR1* gene’s promoter. Male PM carriers have an elevated risk of developing neurological and psychiatric changes, including an approximately 50% risk of the fragile X-associated tremor/ataxia syndrome (FXTAS). The aim of this study was to assess the relationships of regional white matter hyperintensities (*wmhs*) semi-quantitative scores, clinical status, motor (UPDRS, ICARS, Tremor) scales, and cognitive impairments, with *FMR1*-specific genetic changes, in a sample of 32 unselected male PM carriers aged 39–81 years. Half of these individuals were affected with FXTAS, while the non-FXTAS group comprised subcategories of non-affected individuals and individuals affected with non-syndromic changes. The dynamics of pathological processes at the cellular level relevant to the clinical status of PM carriers was investigated using the enzyme AMP-activated protein kinase (AMPK), which is a highly sensitive cellular stress-sensing alarm protein. This enzyme, as well as genetic markers – CGG repeat number and the levels of the *FMR1* mRNA – were assessed in blood lymphoblasts. The results showed that the repeat distribution for FXTAS individuals peaked at 85–90 CGGs; non-FXTAS carriers were distributed within the lowest end of the PM repeat range, and non-syndromic carriers assumed an intermediate position. The size of the CGG expansion was significantly correlated, across all three categories, with infratentorial and total *wmhs* and with all motor scores, and the *FMR1* mRNA levels with all the *wmh* scores, whilst AMPK activity showed considerable elevation in the non-FXTAS combined group, decreasing in the FXTAS group, proportionally to increasing severity of the *wmhs* and tremor/ataxia. We conclude that the size of the CGG expansion relates to the risk for FXTAS, to severity of infratentorial *wmhs* lesions, and to all three motor scale scores. *FMR1* mRNA shows a strong association with the extent of *wmhs*, which is the most sensitive marker of the pathological process. However, the AMPK activity findings – suggestive of a role of this enzyme in the risk of FXTAS – need to be verified and expanded in future studies using larger samples and longitudinal assessment.

## Introduction

The premutation allele of the Fragile X (*FMR1*) gene, containing CGG trinucleotide expansions ranging from 55 to 200 repeats, is known to be associated with a wide range of clinical manifestations depending on sex, age, and the size of the expansion (Loesch and Hagerman, 2011; Hagerman and Hagerman, 2013). Although the pathological mechanisms leading to the premutation-associated conditions are not fully understood, increased *FMR1* transcription has suggested the role of a ‘toxic’ gain of function of the elevated and expanded *FMR1* mRNA (Tassone et al., 2000). More recently, the presence of ‘toxic’ proteins FMRPolyG and FMRPolyA, containing polyglycine (PolyG) and polyalanine (PolyA) tracts, respectively, and resulting from Repeat-Associated Non-AUG (RAN) translation, has been linked to the etiology of these conditions (Todd et al., 2013; reviewed in Boivin et al., 2017). Pathogenic effects have also been attributed to the cellular processes of damage response involved in repair of the loop formations associated with the presence of premutation-size CGG repeat expansions (reviewed by Hagerman and Hagerman, 2013).

This study is concerned with the genetic, cellular and clinical changes occurring in older male carriers of the premutation allele; nearly half of these individuals develop the severe progressive disorder, fragile X-associated tremor/ataxia syndrome (FXTAS), usually after the age of 55 (Hagerman and Hagerman, 2016). The clinical presentation of FXTAS comprises several major (core) features including intention tremor and cerebellar ataxia, and white matter hyperintensities (*wmhs*) in the middle cerebellar peduncles (the ‘MCP sign’) represent a major radiological feature. However, more recent studies have shown a significant prevalence of *wmhs* in the splenium of the corpus callosum (Apartis et al., 2012; Kalus et al., 2016), or in the basis pontis (Loesch et al., 2011) in FXTAS patients. Parkinsonism, cognitive (memory and executive function) deficits, peripheral neuropathy, cerebral *wmhs* and generalized brain atrophy have been listed among the minor features of FXTAS (Jacquemont et al., 2004). Neuropathological findings underlying the brain changes in FXTAS show widespread loss of myelin and axons in cerebellar and cerebral white matter with proportionally lesser gray matter loss, astrocytic pathology, and an abundance of ubiquitin-positive intranuclear inclusions in both neurones and astrocytes which are most frequent in the hippocampus (Greco et al., 2006). Traditionally, the diagnostic criteria for FXTAS relied upon the presence of one major clinical and one major radiological sign (definite), two major clinical signs or one major radiologic and one minor clinical sign (probable), or one major clinical and one minor radiologic sign (possible; Hagerman and Hagerman, 2007). Obviously, evolution from ‘possible’ to ‘definite’ diagnosis as age increases is the most likely scenario.

However, it has been noted that the scope of premutation-associated clinical or radiological changes may extend beyond the syndromic form – that is, FXTAS (Loesch et al., 2005a; Loesch and Hagerman, 2011). The non-syndromic features may include isolated cognitive decline, mood disorders such as depression and/or anxiety, fibromyalgia, or isolated intention tremor; similarly, neurodegenerative changes may extend beyond the major feature of the MCP sign, which occurs in more than a half of FXTAS cases (Brunberg et al., 2002) to involve *wmhs* in supratentorial areas (Loesch and Hagerman, 2011, and present data). Cortical and subcortical gray matter atrophy have been reported in FXTAS (Cohen et al., 2006; Wang et al., 2013c), with an accelerated volume decrease with age in the brainstem (Wang et al., 2017) and in the anterior cerebellar vermis and hemispheres (Hashimoto et al., 2011). These changes, however, appear to be secondary to the white matter disease, which is a major component of brain pathology in FXTAS and beyond, contributing to a considerable brain volume loss (Loesch and Hagerman, 2011; Hagerman and Hagerman, 2013). Several reports based on structural MRI studies have revealed widespread white matter degeneration, which apparently spreads posteriorly from the frontal region with disease progression, but these studies have mainly been limited to male carriers affected with syndromic FXTAS (Wang et al., 2012; reviewed by Brown and Stanfield, 2015).

In this study we investigate the relationship of the level and pattern of involvement of white matter disease and motor and cognitive impairments with *FMR1*-specific genetic changes, in a sample of older males carrying the premutation (PM) allele, with the inclusion of two individuals carrying alleles extending beyond PM into the ‘gray zone’ (GZ) expansion range (45–54 CGG repeats), which have also been associated with FXTAS-like manifestations (Loesch and Hagerman, 2011; Debrey et al., 2016), the risk of parkinsonism in males (Loesch et al., 2009b, 2013), and the elevation of a ‘toxic’ *FMR1* mRNA (Loesch et al., 2007). Half of the carriers included were affected with FXTAS, while the non-FXTAS group comprised subcategories non-affected carriers and carriers affected with non-syndromic changes. We use a semi-quantitative visual *wmhs* rating based on MRI T2/FLAIR imaging to assess the white matter lesions both within and beyond the cerebellar peduncles, including other infratentorial structures and deep hemispheric and periventricular regions, as well as three motor scales and a battery of neuropsychological tests. Furthermore, the dynamics of pathological processes relevant to the clinical status at the cellular level are explored using the enzyme AMP-activated protein kinase (AMPK), assessed in blood lymphoblasts. AMPK is a highly sensitive cellular stress-sensing alarm protein which may be activated by various cellular stresses (Bokko et al., 2007; Hardie et al., 2012). Our results suggest that there may indeed be hyperactivation of AMPK in a sample of non-FXTAS PM carriers compared with the activities of this enzyme in normal controls and carriers affected with FXTAS. Apart from comparative analysis, we assess the relationships of AMPK activity levels, CGG expansion size and *FMR1* mRNA levels, with the extent of the white matter lesions and the severity of clinical involvements.

## Materials and Methods

### Sample

The study was approved by the La Trobe University Human Ethics Committee (No. 01/85). All participants gave informed consent for their involvement. The new sample, recruited and tested between the years 2012–2016, consisted of 24 Caucasian male PM carriers, and two borderline PM/GZ carriers (later referred to collectively as ‘PM carriers’). Out of the total of 26 participants, 12 subjects had FXTAS spectrum diagnosis based on at least two core signs (of whom one individual was classified as FXTAS only on the basis of MCP sign associated with dementia). Eight subjects (later referred to as ‘OTHERS’) manifested other disorders or non-syndromic changes outside the FXTAS spectrum, including: fibromyalgia (1), dementia (2), isolated intention tremors (3) that were associated with minor *wmh* changes but no MCP sign, and panic disorder with depression (1), and orthostatic tremor (1). Six asymptomatic carriers were not affected and are later referred to as ‘Non-affected.’

In addition, we included the data from six carriers with CGG repeats within the PM range, previously recruited and tested by us in 2004–2008, comprising four FXTAS-affected individuals, one carrier manifesting mild ataxia with minor white matter changes (included in the ‘OTHERS’ subcategory), and one non-affected carrier. Altogether we included 16 FXTAS, and 18 non-FXTAS (nine in ‘OTHERS,’ and seven in ‘Non-affected’ groups) totaling 32 PM allele carriers, though some data were missing in a proportion of individuals from each of the three groups. The age range was 50–81 years for the FXTAS group, 39–69 years for the ‘affected with non-syndromic changes’ group, and 58–64 years for the non-affected subgroup, with mean values of 63.0, 61.8, and 53.7 years, respectively. Premutation CGG repeat sizes ranged from 56 to 160, and the two individuals with GZ mutations had 45 and 54 repeats, respectively. The *FMR1* mRNA levels ranged from 2.32 to 5.38, relative to the baseline norm of 1.00 (Tassone et al., 2004). All the carriers were recruited from fragile X families, who were identified through clinical admissions of children with the fragile X syndrome to the Victorian Clinical Genetics Services (VCGS) at the Royal Children’s Hospital in Melbourne, with cascade-testing.

In addition, 21 healthy age-matched non-carrier controls were included for comparison of AMPK levels.

### Neuroimaging Assessments

MRI scans were performed on 1.5 Tesla Siemens or General Electric scanners and included turbo spin-echo 2 dimensional (i) proton-density with T2 weighting and/or (ii) fluid-attenuated inversion recovery (FLAIR) axial images with a 5 mm slice thickness or 3D FLAIR with 1 mm voxel size.

#### Visual White Matter Hyperintensities (*wmhs*) Rating

The extent and severity of supratentorial and infratentorial deep white matter hyperintensities (‘DWMH’), and periventricular white matter hyperintensities (‘PV-WMH’) were evaluated from the proton-density/T2 and/or the FLAIR images by an experienced neuroradiologist (NT) using a visual semi-quantitative method. The evaluation was performed blinded to clinical data, and it was repeated 1 week apart. The DWMH rating was based on the method described by Wahlund et al. (2001). Since DWMH and PV-WMH are likely to result from different pathological processes and vary in extent and severity between different clinical scenarios (van Straaten et al., 2008), and review of previous studies (Brunberg et al., 2002; Rivera et al., 2010) demonstrated increased PV-WMH in FXTAS subjects compared with normal controls, PV-WMH were separately rated as described by other authors (Scheltens et al., 1993; van Straaten et al., 2008). DWMHs were defined as areas of increased T2 signal >3 mm in diameter, and were rated in five regions for each side of the brain: frontal, parieto-occipital, deep temporal and subcortical white matter, and infratentorial regions. The basal ganglia (BG) region, which included the deep nuclei and capsules, was not included in the ratings. Infratentorial included *wmhs* in the MCP and adjacent deep white matter of the cerebellar hemispheres, which is one of the major criteria for the diagnosis of FXTAS. PV-WMHs were defined as confluent hyperintensities adjacent to the frontal or occipital horns (caps) or the bodies (bands) of the lateral ventricles. When PV-WMH were >10 mm they were given a score of two, and any excess was included in the DWMH score. Respective scores were totaled to give: total supratentorial-DWMH (‘Total Supra-DWMH’), total infratentorial DWMH (‘Total Infra-DWMH’), and total periventricular WMH (‘Total PV-WMH’) scores, and the sum of these three regional summary scores was labeled as ‘Total-WMH.’ Chronic lacunes, which were identified as well-defined areas >3 mm with signal characteristics similar to cerebrospinal fluid, were rare and were not included in either the *wmhs* rating or in other analyses. The detailed description of the measures used, together with illustration of the spectrum of changes, can be found in our earlier publication (Trost et al., 2014).

### Neurological and Neuropsychological Assessments

Structured medical history and standard neurological motor rating scales with established inter-rater reliabilities (Richards et al., 1994; Storey et al., 2004; Stacy et al., 2007) were conducted by two neurologists (ES and DZL) with relevant experience in these scales from previous studies. Motor rating scales consisted of the Unified Parkinson’s Disease Rating Scale Part III-Motor (UPDRS-III; Fahn et al., 1987), the International Cooperative Ataxia Rating Scale (ICARS; Trouillas et al., 1997), and the Clinical Rating Scale for Tremor (Fahn et al., 1993).

The Vocabulary and Matrix Reasoning subtests of the Wechsler Adult Intelligence Scale (Third Edition; WAIS-III) were used to calculate a prorated Full Scale IQ score (Wechsler, 1997). Additional WAIS-III subtests were also used: Similarities and Matrix Reasoning subtests represent verbal and non-verbal reasoning, respectively, and thus would also be conceptualized as measures of executive functioning, as would WAIS-III Digit Span (total score) which was employed as a measure of attention and working memory (Wechsler, 1997). The Symbol Digit Modalities Test (SDMT; total score) was used as a measure of information processing speed (Smith, 1982). Raw scores were used in all analyses, except for prorated IQ, as this variable is already adjusted for age.

### Molecular Assessments

#### CGG Repeat Size

The data on CGG repeat expansion size, already available to us from previous diagnostic testing of fragile X families at the VCGS, was assessed using PCRs and Southern Blot analyses, with all assays fully validated by internal and external quality assessment to provide a precision of ±one to two repeats (Khaniani et al., 2008; Loesch et al., 2009a).

#### RNA Isolation and *FMR1* mRNA Expression Levels

This assay was conducted at the MIND Institute, University of California Davis Medical Center, Sacramento, CA, United States. Total RNA was isolated from 3 ml of blood collected in Tempus tubes (Applied Biosystems, Foster City, CA, United States) or from 1 × 10^6^ cells using Trizol (Life Technologies, Carlsbad, CA, United States). The measurement of *FMR1* mRNA expression levels was carried out by quantitative Real Time qRT-PCR on totRNA using custom-designed Taqman gene expression assays (Applied Biosystems) as previously described (Tassone et al., 2004).

#### AMPK Activity

These assays were performed as described by us previously (Annesley et al., 2016). Lysates were prepared from confluent cell lines (∼25 ml) grown in T75 flasks, harvested, lysed in lysis buffer supplemented with phosphatase inhibitors (50 mM Tris-HCl pH 7.4, 150 mM NaCl, 1 mM EDTA, 1 mM EGTA, 1% Triton X-100, 50 mM NaF, 5 mM sodium pyrophosphate) then snap-frozen in liquid nitrogen. Thawed lysates were cleared by centrifugation at 10,000 ×*g* for 5 min. Supernatant total protein concentrations were determined with the Pierce^TM^ BCA Protein Assay Kit (Thermo Fisher Scientific). To concentrate the AMPK protein, one mg of total protein was used in an immunoprecipitation experiment using rabbit polyclonal anti-AMPKα1 antibody α1-(339–358) (Stapleton et al., 1996) bound to equilibrated protein A-agarose beads. The beads were recovered and washed four times by centrifugation before being resuspended in 60 μl wash buffer (50 mM HEPES pH 7.4, 150 mM NaCl, 10% glycerol, 0.1% Tween-20). This was named the AMPK slurry. AMPK activity was assayed over 10 min at 30°C by adding 20 μl of the AMPK slurry to 15 μl buffer (5 mM MgCl_2_, 50 mM HEPES pH 7.4, 0.1% Tween-20, and 1 mM DTT) containing 100 μM SAMS synthetic peptide (NH2-HMRSAMSGLHLVKRR-COOH). Reactions were started by adding [γ-32P]-ATP (final concentration 200 μM) and stopped by spotting 21 μl onto P81 ion-exchange chromatography paper (Whatman, GE Healthcare). Liquid scintillation counting (Perkin Elmer) was used to measure the incorporation of 32P into the SAMS peptide. Duplicates were averaged and normalized against the average value from all the control cell lines, in each independent experiment.

### Statistical Analysis

**FIGURE 1:**
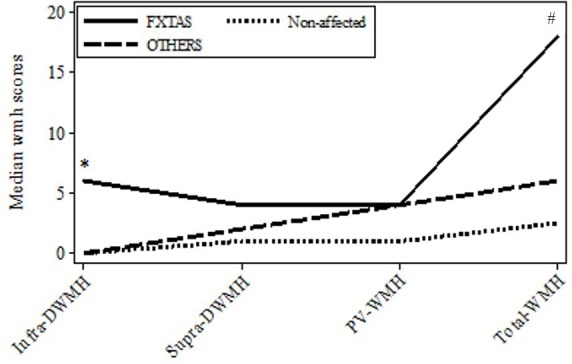
Plot of medians for the major *wmhs* scores in individual brain regions in the three groups: FXTAS, OTHERS, and Non-affected male carriers of premutation alleles. Infra-DWMH, infratentorial *wmhs*; Supra-DWMH, sum of frontal, parieto-occipital and temporal *wmhs*; PV-WMH, periventricular wmhs; Total-WMH, sum of *wmhs* in all five regions. *^∗^p*-value (two-sided) <0.05 for comparison between FXTAS and combined non-FXTAS, and between FXTAS and OTHERS for total infra DWMH. #*p*-value (two-sided) <0.05 for comparison between FXTAS and Non-affected for Total WMH.

**FIGURE 2:**
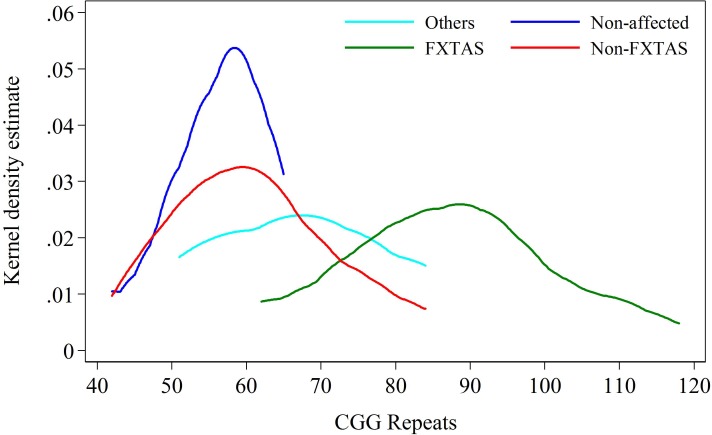
Kernel density distribution for FXTAS and combined Non-FXTAS group, and for OTHERS and Non-affected groups against CGG repeat size.

Comparisons of individual MRI scores between the two non-FXTAS (‘OTHERS’ and ‘Non-affected’) and the FXTAS groups (as in Figure 1), and of activated AMPK levels between FXTAS, the two non-FXTAS groups combined and normal controls (as in Table 2) were conducted using the non-parametric Mann–Whitney test, and ANOVA, respectively. The distribution of CGG repeats and activated AMPK levels was estimated for each of the three groups separately (as in Figures 2, 4, respectively) using a non-parametric kernel density estimation method. The method of least squares was used to assess the relationship between each cognitive and motor score, and CGG size and *FMR1* mRNA levels, and between AMPK activity and genetic and major clinical and *wmhs* measures. If outliers were present, robust regression was employed to minimize the effect of the outstanding observation on the estimated regression coefficients. Although the PM and control participants had been group-matched for age, we tested for the presence of any age effect on individual measures, and the appropriate adjustment was introduced in the regression model if the age effect was significant. In estimating significance level of some of the relationships we used a one-sided test, which tests the probability of the relationship in one direction disregarding the possibility of a relationship in other direction, and thus provides more power to detect an effect This was applied in testing the effect of genetic changes (CCG expansion and mRNA levels), which are causative of clinical pathology represented by neurological changes and white matter lesions, so that the direction of the relationships (if any) is entirely predictable. A two-sided test was applied in the relationship of AMPK with the clinical or genetic status, where we tested the possibility of the relationship in both directions, and in *wmhs* comparisons between the carriers’ groups.

**FIGURE 3:**
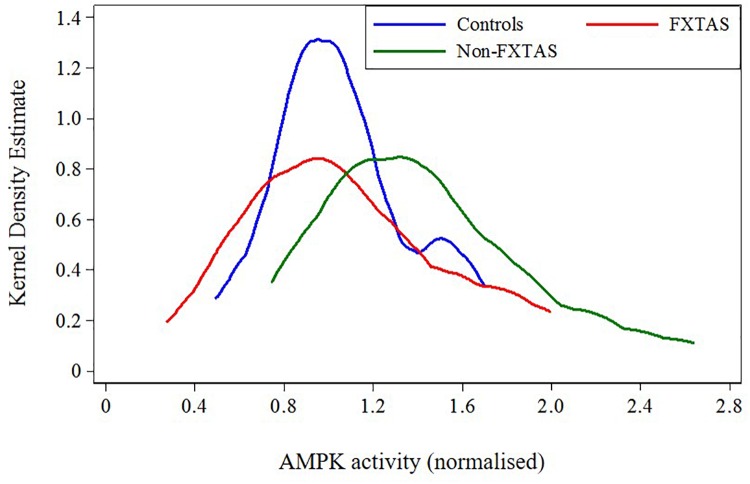
Plot of distribution of AMPK activity for FXTAS, Non-FXTAS PM carriers, and Controls without CGG expansions.

**FIGURE 4:**
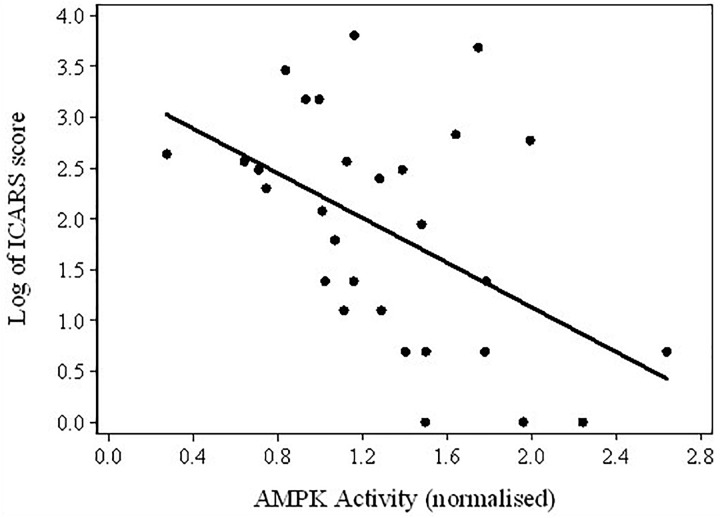
Scatterplot of the log-transformed ICARS scores versus AMPK activity in the total sample of PM carriers.

To adjust for multiple testing, we used the false discovery rate control (FDR), which is a recommended alternative to Bonferroni-type error adjustments, and is a more suitable approach for drawing statistical inferences in our study (Glickman et al., 2014). Although the Bonferroni correction is better known and used more frequently, it tests a composite null hypothesis about the data based merely on the number of tests, at the cost of testing the significance of individual component hypotheses; while FDR considers a distribution of the *p*-values across individual tests, as well as their number, and ensures greater power, at the cost of some underestimation of FDR. All analyses were performed using STATA statistical software (version 13).

## Results

### Sample Characterization

Since white matter degeneration has been considered the most prominent feature of brain pathology associated with PM alleles and often precedes clinical manifestations of FXTAS, we first ascertained if the degree and/or location of this process differs between the three initially distinguished clinical groups comprising subjects diagnosed with FXTAS, those affected with non-syndromic changes (‘OTHERS’),’ and not affected carriers (‘Non-affected’). Data in Figure 1 show the distributions of median *wmhs* scores in individual brain regions separately for each of these three groups. The intermediate position of the OTHERS group in relation to the other two groups with respect to the total *wmhs* (WMS) score is noticeable, although differences between small samples in the remaining regional *wmhs* scores were not significant.

The intermediate position of the OTHERS group is more evident when all three clinical categories of carriers are distributed in relation to the CGG repeats within the PM/upper-end GZ range. The data in Figure 2 show that the distribution for carriers in the Non-affected group peaks at the level of 55–60 CGGs, the distribution for individuals with FXTAS ranges between 65 and 120 CGGs (peaking at ∼85 repeats), and the carriers in the OTHERS group assume an intermediate position with some overlap with each of the other two groups. Although the intermediate position of the group representing non-syndromic pathologies is an important finding, the sizes of both non-FXTAS groups are too small to be considered separately. They were therefore combined into a ‘Non-FXTAS’ group for statistical analysis. Distributions of individuals in FXTAS and Non-FXTAS groups against CGG repeat size shows that the latter peak at ∼60 repeats, also showing some overlap with the FXTAS group.

### Relationship of Individual Motor and Cognitive Impairments to CGG Repeat Size and *FMR1* mRNA Levels

The regression models were applied to the total sample, as all participants are linked by a common recruitment criterion: carrier status of the premutation allele. The models were also applied to the FXTAS group separately, in which participants are linked by their clinical diagnosis. The results shown in Figures 1, 2 already suggested that there is a relationship between clinical manifestations/diagnostic groups and CGG repeat size within the premutation range on the one hand, and these manifestations and regional white matter lesions, on the other. We expanded on these results by assessing the relationships of all individual *wmhs* scores, and the motor and neuropsychological scores included in the study and listed in Table 1, with genetic predictors: the size of CGG, and the levels of *FMR1* mRNA. The results show significant associations, in both total and FXTAS samples, between the combined *wmhs* (Total-WMH) and infratentorial *wmhs* (Infra-DVMH) scores and CGG repeat size; however, a significant correlation between periventricular *wmhs* (PV-WMH) scores and CGG size in the FXTAS sample did not survive after adjustment of the significance level for multiple comparisons. The relationship of all three motor scores with CGG size remained significant after adjustment for multiple comparisons. In the total sample, amongst the six cognitive scores, the relationship between CGG repeat size and Prorated IQ, Similarities and Digit Span remained significant after adjustment for multiple comparisons, while in the FXTAS group, the relationship between CGG repeat size and Prorated IQ, Similarities and SDMT also remained significant after adjustment. However, after applying a Bonferroni-type of adjustment, the relationship of CGG repeat size with cognitive scores was statistically significant only for Similarities, but remained significant for all motor and *wmhs* scores (except PVWMH).

**Table 1:** Relationship between each cognitive and motor score with CGG repeat numbers and FMR1 mRNA levels, in the total sample of premutation carriers and in the FXTAS group.

	CGG						*FMR1* mRNA					
	Total carriers			FXTAS			Total carriers			FXTAS		
	*N*	Coef	*p*-Value	*N*	Coef	*p*-Value	*N*	Coef	*p*-Value	*N*	Coef	*p*-Value
**MRI scores**												
Supra-DWMH+	29	0.02	0.298	16	0.022	0.3715	17	1.969	**0.0070^∗^**	13	2.83	**0.0070^∗^**
Infra-DWMH+	29	0.159	**<0.0001**^∗^	16	0.199	**<0.0001^∗^**	17	2.911	**0.0010^∗^**	13	2.973	**<0.0001^∗^**
PV-WMH	29	0.035	0.131	16	0.105	**0.0225**	17	2.122	**0.0001^∗^**	13	2.466	**0.0015^∗^**
Total-WMH	29	0.225	**0.0080^∗^**	16	0.485	**0.0002^∗^**	17	5.778	**0.0005^∗^**	13	7.717	**0.0003^∗^**
**Motor scores**												
UPDRS	28	0.123	**0.0005**^∗^	14	0.052	0.1885	15	0.023	0.493	11	0.853	0.281
ICARS	28	0.335	**0.0002**^∗^	14	0.246	**0.004^∗^**	15	1.142	0.381	11	1.947	**0.037**
Tremor scale	22	0.283	**0.0030**^∗^	10	0.071	0.3395	9	6.54	0.279	7	-1.42	0.472
**Cognitive scores**												
Prorated IQ	30	-0.281	**0.028^∗^**	16	-0.209	**0.037**	17	0.401	0.4565	13	-1.664	0.3575
Similarities	30	-0.106	**0.002^∗^**	16	-0.08	**0.016^∗^**	17	-0.016	0.492	13	-0.273	0.404
Vocab	26	-0.054	**0.025**	14	-0.047	0.1145	12	-0.566	0.2015	12	-0.547	0.227
Matrix reasoning	26	-0.06	**0.033**	14	-0.008	0.4405	14	0.307	0.365	12	0.116	0.4625
Digit span	26	-0.027	**0.013^∗^**	14	0.025	0.3115	13	-0.113	0.4305	12	-0.591	0.2525
SDMT RS	15	-0.434	**0.023**	8	-0.146	**0.014^∗^**	10	2.17	0.269	8	-1.044	0.4425

*p-Values were for one-sided test and ^∗^< 0.05 after adjustment for multiple comparisons. +Age adjustment applied. Bold figures indicate p-values < 0.05 before adjustment for multiple comparisons.*

When *FMR1* mRNA levels were used as the predictor, highly significant associations between the level of this transcript and all *wmhs* scores were seen in both FXTAS and Total samples, before and after adjustment for multiple comparisons. However, a significant association between *FMR1* mRNA level and ICARS score in the FXTAS group did not survive adjustment for multiple comparisons.

### AMPK Activity – A Novel Blood Biomarker in Relation to Clinical and PM Status Categories

Because of the highly variable nature of assayed AMPK activity and the small sample sizes, we combined previously distinguished categories: OTHERS and Non-affected carriers into the ‘Non-FXTAS’ group in both descriptive and regression analyses. The position of each of these two groups relative to normal controls without any CGG premutation expansion (‘Controls’) with respect to AMPK activity levels shows a shift of these levels toward higher values in the Non-FXTAS compared with the Controls (Figure 3).

The results of statistical comparison between FXTAS, Non-FXTAS, and Control samples (Table 2) show a highly significant elevation of AMPK activity levels in the Non-FXTAS group compared with Controls (*p* = 0.006). In the FXTAS group, the mean value is considerably lower compared with the Non- FXTAS group (*p* = 0.038), and it is not significantly different from that in the Control sample (*p* = 0.949). Two-sided test for the *p*-values has been considered because a direction of change could not be anticipated.

**Table 2:** Descriptive statistics for AMPK activity.

	*N*	Mean	*SD*
Control	21	1.07	0.33
FXTAS	14	1.08	0.47
Non-FXTAS	18	1.45	0.48

*p-Value = 0.0143 for comparison between the means (ANOVA).*

Considering the results of these comparisons, it was of interest to test the assumption that the fall in AMPK activity levels between Non-FXTAS and FXTAS groups corresponded to the appearance or increase in severity of tremor/ataxia – the most typical neurological changes in FXTAS – as measured by the ICARS scores. The results of regression between these scores and AMPK activity provided evidence for a highly significant relationship (*coefficient* = -1.10, *p*-value = 0.009), and the scatterplot shows a continued upward trend in ICARS scores with a downward trend in AMPK activity levels, in spite of the extensive variability in the latter values (Figure 4).

Similar results were obtained for correlation between AMPK activity levels and the extent of *wmhs* lesions in the brain: the relationship between Total WHM and AMPK activity was significant for the raw values (*coefficient* = -7.95, *p* = 0.026), and highly significant for the (square root) transformed AMPK values (*coefficient* = -1.68, *p* = 0.009). In addition, amongst all five *wmhs* measures (as in Table 1) included in the relationship analysis with AMPK activity, two (Total-WMH and Supra-DWMH) representing the sum of regional *wmhs* and supratentorial deep white matter *wmhs*, respectively, remained significant after adjustment for multiple comparisons (*coefficients* = -7.945 and -3.371, respectively, with *p* = 0.026 for both regressions). However, AMPK activity was not significantly correlated with the two genetic biomarkers, CGG expansion size and *FMR1* mRNA levels.

## Discussion

This is the first study to encompass analysis of genetic and cellular blood biomarkers in relation to each other and to neurological and cognitive status in an unselected sample of older male carriers of the *FMR1* premutation allele. Although FXTAS is a major disorder linked to this allele, we draw attention here to a subgroup of non-FXTAS PM carriers with non-syndromic features, such as isolated tremor, cognitive decline, fibromyalgia and mood disorders, in association with non-syndromic or even occasionally typical *wmhs* locations. Since the neural pathology in premutation carriers is linked to a dynamic mutation, implying continuity of genetic effects, it was of interest to establish the position of the three clinical groups relative to each other, and of the pooled non-FXTAS group – comprised of the non-affected and non-syndromic groups combined – to the FXTAS group in relation to the distribution of CGG repeat sizes. Our data shows that while the non-affected carriers were positioned at the lowest end of the premutation range, including the values bordering with the gray zone range, the individuals affected with non-syndromic changes were positioned, with respect to CGG sizes, between the asymptomatic and FXTAS groups, with the latter peaking at ∼85 CGGs.

This finding is of interest considering the earlier results based on neuroblastoma-derived human cell cultures, which showed that a CGG-repeat size threshold of neural toxicity lies between 62 and 95 repeats (Hoem et al., 2011). Moreover, the results of the same study showed a disparity between *FMR1* mRNA concentration and cell viability at the lower end of the CGG size distribution in the premutation range of 55–200: the high levels of apparently ‘toxic’ mRNA below the threshold of the ‘toxic’ CGG size did not induce cellular toxicity. At the same time, expression of 95-CGG-repeat mRNA led to reduced cell viability, which became more pronounced with increasing RNA expression. Our present data suggesting that the significant relationship between CGG size and *FMR1* mRNA levels might occur exclusively in the FXTAS group, is in concert with the above findings. While the Spearman correlation between CGG repeat size and *FMR1* mRNA levels (ρ = 0.64) was significant (*p* = 0.019) in the present sample of 12 FXTAS patients, with the scatterplot showing a close linear relationship, it was negligible in a sample of four non-FXTAS individuals – the result being inconclusive because of small sample size. However, this clearly indicates the need to conduct an analysis along the same lines in larger samples, especially of the non-FXTAS carriers, since the confirmation of this preliminary observation may contribute to an explanation of the nature and effects of CGG repeat size in relation to mRNA -mediated toxicities, including the more recently discovered RAN translation occurring with *FMR1* premutation alleles (Todd et al., 2013; Boivin et al., 2017). Our new data combined with the historical findings suggest that concerted action between these two aspects of genetic pathophysiology characterizing the PM alleles may be conducive to conversion from the non-affected, or affected with non-syndromic changes state, to fully-manifest FXTAS in some carriers, while in some others the non-syndromic manifestations may represent a different form of PM-associated condition from FXTAS, not progressing to FXTAS, occupying an intermediate position between asymptomatic and FXTAS groups with respect to CGG expansion size, and not displaying the typical infratentorial *wmhs* lesion of FXTAS.

Regression analysis revealed relationships of CGG repeat numbers and *FMR1* mRNA levels with the motor and *wmhs* measures, and with major cognitive scores. The analysis was conducted in the FXTAS group, whose subjects have a clinical diagnosis in common, as well as in the total PM sample, whose subjects are linked by a common recruitment criterion: carrier status of the PM allele. The size of the CGG expansion showed highly significant correlations with all the motor scores, infratentorial and total *wmhs.* The relationships with the aspects of cognitive tests consistent with the established pattern of cognitive impairments in FXTAS (reviewed by Grigsby et al., 2014) are less obvious, however, and need to be confirmed in a larger, independent sample, considering the possibility of type I errors in the process of FDR control. In an earlier study of unselected PM carriers (equivalent to our ‘Total carriers’ in Table 1), Leehey et al. (2008) reported the relationship between CGG expansion size and a single combined measure using the three motor scales used in this study. Several other reports were limited to an association between brain volume loss and CGG repeat size in a sample of older male PM carriers (Loesch et al., 2005b) and in a sample of FXTAS males (Cohen et al., 2006; Wang et al., 2017).

Earlier attempts to relate *FMR1* mRNA levels to phenotypic data in PM carriers have been few, however, and are not readily comparable to the present study (see Loesch and Hagerman, 2011, for review). This may be because, while there is a close correspondence in the size of the CGG expansion between blood and brain cells (Tassone et al., 1999), there are discrepancies between the levels of mRNA transcript between blood and brain (Tassone et al., 2004). Nevertheless, our data provide evidence for the relationships between phenotypic changes and elevated *FMR1* mRNA levels in PM carriers. These relationships were strong but limited to the total and the three regional (Supra-DWMH, Infra-DWMH, and PV-WMH) *wmhs* included in this analysis; the only clinical feature showing significant relationship after adjustment for multiple comparison was prorated IQ. This result should not be surprising since white matter lesions (as revealed by MRI) appear earlier than clinical features. Indeed, subtle changes in the integrity of white matter (MRI fiber tract changes) may occur in male carriers without, or prior to, the occurrence of FXTAS (Battistella et al., 2013; Wang et al., 2013a,b,c).

Notably, our findings that the relationships between both genetic markers and the clinical/radiological changes were significant in the Total PM sample, as well as in the FXTAS group, suggest a progressive effect of increasing *FMR1* CGG expansion size and level of expression of ‘toxic’ mRNA on the type and severity of neurological manifestations across the whole spectrum of PM carriers. This effect, may therefore generate mild and/or atypical manifestations such as encountered in our non-syndromic (‘OTHERS’) category. Another novel finding from the present study is that the genotype-phenotype relationships extend beyond the middle cerebellar peduncles and the adjacent white matter – the region that has been considered a major target for *FMR1* mRNA toxicity and highly relevant to the clinical manifestations of FXTAS (Hagerman and Hagerman, 2013, 2016).

All the above data further illuminates the still unsolved problem of a diversity of clinical manifestations in PM carriers ranging from normality to the most severe involvement in the form of FXTAS, which must clearly depend on the final effect of interplay, in individual carriers, between cellular stresses including toxicity of *FMR1* mRNA, linked with the chain of stressful biochemical changes, and cellular stress responses. In an attempt to make the first step toward understanding this process, we chose to relate clinical status and the measures of neurological involvement in PM carriers to the levels of activated AMPK – a highly sensitive cellular stress response protein (Hardie et al., 2012). Although these levels were assessed in cultured lymphoblasts, the only tissue available to us for research in living subjects, our results provide indirect evidence for this marker’s relevance to neurological involvement in PM carriers by showing a significant (inverse) relationship of activated AMPK levels with the severity of tremor/ataxia (assessed by the ICARS), and some *wmhs* measures representing a degree of white matter pathology, across all three clinical categories of these carriers. AMPK activity is not the only biochemical change in PM lymphoblasts that has been found to correlate with the severity of neurological involvement. We have reported previously that mitochondrial respiratory function in these cells (rates of basal and maximal uncoupled O_2_ consumption, ATP synthesis and Complex I activity) is positively correlated with *wmhs* and ICARS in these carriers (Loesch et al., 2017).

In the present study we observed a significant elevation of AMPK activity in the non-FXTAS group compared with the levels in healthy controls and FXTAS patients, as well as, consistent with this result, negative correlations between the level of this activity and the ICARS and *wmh* scores. If these findings are confirmed in larger samples, they will suggest, in the light of earlier data (Herrero-Martın et al., 2009; He et al., 2014; Wu et al., 2014; Distelmaier et al., 2015; Hardie, 2015; Xu et al., 2017) that AMPK activity in PM carriers may be predominantly protective against stress-related cellular damage, thereby restraining the development of mitochondrial dysfunction as seen in FXTAS (Ross-Inta et al., 2010; Napoli et al., 2011). Notably, AMPK has been shown to play cytoprotective roles in diverse cell types, including neuronal cells, cardiomyocytes, fibroblasts, and epithelial cells. However, only longitudinal studies in larger samples, considering the three clinical groups as distinguished in our study, can provide direct evidence that the elevated AMPK activity in response to cellular stresses in different individuals is a disease modifier, so that PM individuals with higher levels of activity are protected, while individuals with lower levels of activity are more susceptible to the development of FXTAS. Such future studies would also enable testing of an alternative hypothesis: that the observed elevation of AMPK activity in non-FXTAS PM carriers is a predisposing rather than preventative factor through the pathways leading from chronic elevation of this enzyme to mitochondrial dysregulation and neurodegeneration While our preliminary results regarding AMPK levels and correlations are ambiguous, they may bring us a step closer to understanding the pathomechanisms of FXTAS development: rather than implying a lack of meaningful link between activated AMPK levels and clinical phenotype, the higher levels of activated AMPK, exclusively in the non-FXTAS group compared with normal controls (as in Table 2), may be interpreted as suggestive of its protective role, by preventing progression of non-FXTAS to FXTAS; whilst the fall of the AMPK levels from non-FXTAS to FXTAS (as shown by data in Figure 4 and Table 2) may, alternatively, reflect its damaging role by accelerating neuronal changes toward the FXTAS phenotype.

Apart from small sample sizes, an apparent limitation of this study is our use of lymphoblasts for functional molecular tests. However, lymphoblasts have been used successfully to study the roles of FMR1 mRNA, antisense RNA and FMR1 protein expression in PM carriers, FXTAS and Fragile-X patients (Ladd et al., 2007; Hagerman and Hagerman, 2013), and the present study provides unequivocal evidence for the relationship of the FMR1 mRNA levels assessed in the lymphoblasts and the severity of the white matter lesions. Although AMPK activities have not been studied previously in lymphoblasts from PM carriers, the relevance of AMPK expression in blood cells to neurodegenerative diseases as a biomarker and a potential molecular target in these disorders has recently been reviewed (Peixoto et al., 2017).

Furthermore, lymphoblasts have been used successfully to study underlying pathomechanisms in other diseases involving toxic gain of function RNA or protein aggregates and RAN translation. Thus, RAN translation producing toxic dipeptide repeats was measured in lymphoblasts of patients with C9orf72-associated ALS and frontotemporal dementia (Liu et al., 2014; Mizielinska and Isaacs, 2014; Niblock et al., 2016).

Most importantly, our own results concerning AMPK have shown, for the first time, the relevance of this marker in any human tissue to PM-associated neurological status. However, we still recommend that this finding should be supported by studies, in larger samples, using the lymphoblasts, as well as other tissues available in living humans, or neural cell tissue in an experimental model. This is especially important since our results suggest that AMPK may be developed as a prognostic biomarker/and/or treatment target. Therefore, we plan to use the AMPK activity test in cultured fibroblasts from male carriers from different clinical categories and/or at different stages of the clinical progression. In conclusion, our study has identified specific links between two major genetic markers and one cellular stress marker, and the level of neurological involvement – both clinical and radiological – in an unselected sample of older male PM carriers. This sample was divided into three categories depending on the clinical status of the PM carriers, and we placed particular emphasis on the nature of this involvement in the non-FXTAS group, and of a transition from non-affected or non-syndromic status to full-blown FXTAS, and the underpinning neurodegenerative process. Although the small sample size did not allow for consideration of this non-syndromic subgroup separately in some statistical analyses, our data suggests a link between lower CGG repeat expansion size within the premutation range and the occurrence of non-syndromic neurological changes. Further investigation based on a longitudinal study of larger cohorts will be necessary to establish what proportion of these subjects represents preclinical stages of FXTAS, and what proportion remains in the non-syndromic clinical category.

Questions about the mechanism by which AMPK is dramatically elevated in the non-FXTAS carriers compared with healthy controls and normalizes in the fully syndromic FXTAS group should be explored in larger samples, including all three clinical groups, as distinguished here, in a longitudinal study. If the role of AMPK is confirmed and the mechanism is determined, this will open a new avenue of research into the dynamics of the effects of PM alleles on cellular functions and pathology, as well as providing a new biomarker of clinical status, and a potential disease-modifying/preventative target.

## Ethics Statement

This study was carried out in accordance with the recommendations of the National Statement on Ethical Conduct in Human Research (2007), National Health and Medical Research Council. The protocol was approved by the La Trobe University Human Ethics Committee. All subjects gave written informed consent in accordance with the Declaration of Helsinki.

## Author Contributions

DL and ES contributed conception, design, organization and partial execution of the study, neurological assessments, and motor scale scoring. DL wrote the first draft of the manuscript. ES supervised neuropsychological testings and contributed to writing the final draft of the manuscript. NT interpreted magnetic resonance images and scored regional white matter hyperintensities. MB designed and executed statistical analysis. EH conducted neuropsychological testing. SL, SA, OS, CA, DF, and PF organized and executed major aspect of the project: setting up/maintaining cell cultures, extracting DNA and assessment of CGG repeats. PF contributed to writing the final version of the manuscript, supervised tissue preparation and testing, and contributed to the interpretation of AMPK results. FT conducted RNA analysis and contributed to writing the final version of the manuscript. Z-PC and BK conducted AMPK assays. BK supervised AMPK testings, contributed to interpretation of AMPK results and writing the final version of the manuscript. All authors contributed to manuscript revision, read and approved the submitted version.

## Conflict of Interest Statement

The authors declare that the research was conducted in the absence of any commercial or financial relationships that could be construed as a potential conflict of interest.
